# Contrastive self-supervised representation learning without negative samples for multimodal human action recognition

**DOI:** 10.3389/fnins.2023.1225312

**Published:** 2023-07-05

**Authors:** Huaigang Yang, Ziliang Ren, Huaqiang Yuan, Zhenyu Xu, Jun Zhou

**Affiliations:** ^1^School of Computer Science and Technology, Dongguan University of Technology, Dongguan, China; ^2^CAS Key Laboratory of Human-Machine Intelligence-Synergy Systems, Shenzhen Institute of Advanced Technology, Chinese Academy of Sciences, Shenzhen, China

**Keywords:** human action recognition, multimodal representation, feature encoder, contrastive self-supervised learning, Transformer

## Abstract

Action recognition is an important component of human-computer interaction, and multimodal feature representation and learning methods can be used to improve recognition performance due to the interrelation and complementarity between different modalities. However, due to the lack of large-scale labeled samples, the performance of existing ConvNets-based methods are severely constrained. In this paper, a novel and effective multi-modal feature representation and contrastive self-supervised learning framework is proposed to improve the action recognition performance of models and the generalization ability of application scenarios. The proposed recognition framework employs weight sharing between two branches and does not require negative samples, which could effectively learn useful feature representations by using multimodal unlabeled data, e.g., skeleton sequence and inertial measurement unit signal (IMU). The extensive experiments are conducted on two benchmarks: UTD-MHAD and MMAct, and the results show that our proposed recognition framework outperforms both unimodal and multimodal baselines in action retrieval, semi-supervised learning, and zero-shot learning scenarios.

## 1. Introduction

Automatic recognition framework is a research field that aims to develop systems capable of identifying and classifying human actions or behaviors, which is to enable machines to understand and interpret human behavior, with applications in areas including video surveillance, healthcare, sports analysis, and human-computer interaction (Li et al., 2016a,b; He et al., 2023). Different techniques in real life adopt different types of data inputs, but each modality has its own advantages and limitations ( Sun et al., 2023). To achieve more robust and accurate feature extraction, some approaches improve the performance of models by aggregating the advantages of various modalities in a reasonable manner. Due to the success of deep learning in the past decades, a large number of ConvNets-based frameworks have made impressive achievements in the field of multimodal visual tasks (Grillini et al., 2021; Mughal et al., 2022; Li et al., 2023). However, most of them require many large amounts of labeled data, especially for multimodal data (Zhang et al., 2019, 2020), and labeling the data requires exponentially more time and effort (Li et al., 2009).

Recently, self-supervised representation learning has made significant progress on visual tasks, which is mainly divided into the pre-training and fine-tuning stages (Chen et al., 2020; Grill et al., 2020). In the pre-training stage, it focuses on constructing feature representations of different views by unlabeled samples. In the fine-tuning stage, these representations are used as inputs and fed into a small-scale linear classifier, which requires only a small amount of labeled data. Moreover, contrastive learning is one of the self-supervised learning, where the core concept is to pull the representation distance between positive samples closer and push the distance away from other negative samples. For example, the CMC framework (Tian et al., 2020) is mainly to form positive samples between different data modalities, and consider other different samples as negative sample pairs. Due to the problem of relying too much on negative sample pairs, it is necessary to set a large batch size or a queue for storing negative samples in the learning process, therefore leads to a complex model and is vulnerable to information collapse.

In order to overcome the above shortcomings, inspired by Barlow Twins and VICReg (Zbontar et al., 2021; Bardes et al., 2022), we propose a contrastive self-supervised learning framework for unimodal and multimodal without relying on negative samples. Our proposed method employs multimodal samples as input data, e.g., skeleton sequence and inertial measurement unit signal (IMU). The main contributions of this paper are as follows:

- A unimodal contrastive self-supervised framework is proposed to encode and learn feature representations for multimodal action recognition with skeleton sequence and IMU data.
- The proposed recognition framework is extended to multimodal contrastive self-supervised learning. The model is designed to obtain simple and efficient feature representations without negative samples.

The remainder of this paper is organized as follows. Section 2 presents an overview of related works. In Section 3, we provided a detailed introduction to the proposed method. Section 4 provides experimental results for benchmark datasets and comparisons with state-of-the-art. Section 5 concludes this paper and look forward to future work.

## 2. Related works

In this section, we discuss unimodal, multimodal, and contrastive learning methods for human action recognition from the perspective of input data modality.

### 2.1. Unimodal human action recognition

Unimodal human action recognition primarily focuses on classifying and recognizing actions by using a single modality, including RGB videos, depth and skeleton sequences, IMU data, etc. This field encompasses tasks such as feature extraction, feature representation, and the construction of deep learning models, including convolution neural networks (CNNs) (Andrade-Ambriz et al., 2022; Islam et al., 2022; Xu et al., 2022), recurrent neural networks (RNNs) (Shu et al., 2021; Shen and Ding, 2022; Wang et al., 2022), graph convolution networks (GCNs) (Cheng et al., 2020; Chi et al., 2022; Feng et al., 2022; Tu et al., 2022) and Transformer models (Chen and Ho, 2022; Mazzia et al., 2022; Ahn et al., 2023).

Since the skeleton sequence would not be sensitive to viewpoint variation and circumstance disturbance, there are numerous skeleton-based methods is developed for human action recognition. In CNN-based methods, Li et al. (2018) proposed an end-to-end convolutional co-occurrence feature learning framework from the perspectives of intra-frame representation and inter-frame representation of skeleton temporal evolutions, which introduced a global spatial aggregation method and discarded the local aggregation approach. In RNN-based methods, Xie et al. (2018) aimed to address the issue of skeleton variations in 3D spatiotemporal space, which proposed a spatiotemporal memory attention network based on RNN and CNN to perform frame recalibration of skeleton data in the temporal domain. Regarding GNN-based methods, Yan et al. (2018) emerged as a classic approach based on spatial-temporal graph convolution networks. The core idea was to model human body joints as graph nodes and the connections between joints as graph edges, and the multiple graph convolutional layers were stacked to extract high-level spatial-temporal features. In Transformer-based methods, Plizzari et al. (2021) model employed a spatial self-attention module to capture intra-frame interactions among different body parts and a temporal self-attention module to model inter-frame correlations.

For IMU data, due to its ability to provide good complementary features and better privacy protection, it is gradually being used for human action recognition tasks. Through convolutional layers and pooling layers, CNN (Yi et al., 2023) were able to capture local and global features in IMU data, extract relationships between skeleton body parts, and achieve accurate classification of different actions. In IMU-based human action recognition, RNN (Al-qaness et al., 2022) utilized their memory units (e.g., Long Short-Term Memory Units or Gated Recurrent Units) to capture the temporal evolution of skeleton sequence, extracting crucial motion patterns and action features from it. Additionally, there have been research efforts that combined the strengths of CNNs and RNNs to comprehensively utilize the spatiotemporal information in IMU data for human activity recognition (Challa et al., 2022; Dua et al., 2023). It is worth noting that, with the progress of research, other IMU-based human action recognition methods have emerged, such as those based on Transformers (Shavit and Klein, 2021; Suh et al., 2023).

### 2.2. Multimodel human action recognition

Due to the limitation of single modal, it is difficult to further improve the performance of recognition model. Since the complementary information provided by different modalities, researchers have become interested in combining multimodal features to improve recognition performance, such as skeleton and IMU data (Das et al., 2020; Khaertdinov and Asteriadis, 2022). There are many excellent recognition models are developed to leverage the strengths of different modalities and achieve more robust and accurate action recognition. However, the main challenge in executing multimodal recognition lies in effectively fuse the feature information from different modalities. Based on the above statement, the related work in multi-modal human action recognition can be roughly categorized into modality fusion and feature fusion, and we focus on the fusion method of skeleton sequence and IMU signal features.

Skeleton data provides precise positional information of human joints, while IMU data provides measurements from sensors such as accelerometers and gyroscopes (Das et al., 2020). By fusing skeleton and IMU data, more comprehensive and rich action features can be obtained. From the perspective of modality fusion, Fusion-GCN (Duhme et al., 2022) directly integrates IMU data into existing skeletons in the channel dimension during data preprocessing. Furthermore, RGB modality is processed to extract high-level semantic features, which are then fed into the GCNs as new nodes for fusion with other modalities. From the perspective of feature fusion (Khaertdinov and Asteriadis, 2022), features from different modalities are combined and integrated to achieve more representative and discriminative representations. In addition, cross-modal contrastive learning networks through knowledge distillation are also an effective identification method. Liu et al. (2021) proposed a Semantics-aware Adaptive Knowledge Distillation Network (SAKDN) that utilizes IMU data and RGB videos as inputs for the teacher and student model, respectively. The SAKDN adaptively fuses knowledge from different teacher networks and transfers the trained knowledge from the teacher network to the student network. The CMC (Tian et al., 2020) framework proposed a multi-modal learning architecture based on contrastive representation learning, which extended the representation learning to multiple modalities for improving the quality of the learned features with the number of modalities increased. It demonstrated the subtle relationship between mutual information across multiple modalities and multiple viewpoints. Similarly, CMC-CMKM (Brinzea et al., 2022) employed cross-modal knowledge distillation to perform feature-level fusion of IMU data and Skeleton information, which has achieved good recognition performance.

### 2.3. Contrastive learning for human action recognition

Recently, several advanced self-supervised learning methods have been proposed with excellent results in image and video tasks. Self-supervised contrast learning focuses on the variation between different views of the same or different samples, and better robust and transferable feature representations can be learned through contrast loss. SimCLR (Chen et al., 2020) incorporated a new contrastive loss function called Normalized Temperature-Scaled Cross-Entropy Loss (NT-Xent) into the network, which is a simple and effective contrastive learning framework. In contrast, BYOL (Grill et al., 2020) designed a more scalable and easily trainable self-supervised learning approach by contrasting the hidden representations in the network. Furthermore, to obtain more distinctive representations without requiring negative samples, Barlow Twins (Zbontar et al., 2021) minimized the correlation between features by employing the Barlow Twins loss. In addition, the biggest advantage of VICReg (Bardes et al., 2022) is its simplicity and effectiveness, which only necessary to compare along the batch dimension by invariance, variance and covariance, and does not require the weights of two branches to be shared.

In the case of action recognition tasks, most of the self-supervised contrastive learning is mainly applied to individual modalities, such as sensor data, skeleton sequence, or RGB video. To date, there has been a large number of works on fully supervised learning for multimodal human action recognition, and the disadvantage of these methods is that they require a large number of labeled samples for training. In contrary, to our knowledge, self-supervised contrastive learning frameworks are rarely used in the field of multimodal human action recognition. Akbari et al. (2021) adopted a convolution-free Transformer architecture to train unlabeled video, audio, and text data end-to-end, and evaluated the model performance through downstream tasks such as video action recognition, audio event classification, image classification, and text-to-video retrieval. Inspired by VicReg (Bardes et al., 2022) and multimodal framework CMC, we propose a simple and effective self-supervised contrastive learning framework based on VICReg to address the multimodal human action recognition problem of IMU and skeleton data.

## 3. Methodology

### 3.1. Problem definition

Multimodal-based action recognition is defined as the fusion of different data modalities to obtain more comprehensive human pose and more precise action information. Specifically, for a given input {*X*^*m*^|*m*∈*M*} from a multimodal set *M*, the goal is to predict the label *y*∈*Y* with the associated input *X*. In our work, we focus on IMU signal data and Skeleton sequences. IMUs could be used to measure the pose and acceleration of the human body with multivariate time series on the x, y and z axes for human motion recognition and analysis. Specifically, for S wearable sensors with S signal channels acquired at any t time stamp, we can define the input signal as $x_{t}=\left[x_{t}^{1},x_{t}^{2},\ldots ,x_{t}^{S}\right]\in {\mathbb{R}}^{S}$. Therefore, the IMU modal inputs are represented in matrix form as $X^{i}=\left[x_{1},x_{2},...,x_{T}\right]\in {\mathbb{R}}^{T\times S}$ for any *T* time stamp. Furthermore, skeleton sequences can be collected by a pose estimation algorithm or a depth camera, which contain several joints of a human body, and each joint has multiple position coordinates. For a given skeleton sequence *X*^*s*^∈ℝ^*C*×*T*×*V*^, as 2D coordinates are used, the input channel *C* = 2, *T* denotes the number of frames in a sequence, and V means that the number of joints with respect to the dataset collection method.

### 3.2. Feature encoder

**Figure 1:**
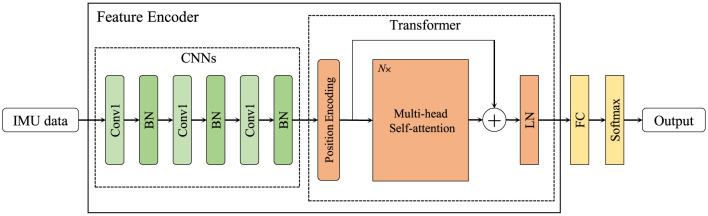
The feature encoder for IMU data. “BN” denotes batch normalization, “LN” indicates layer normalization, and *N*× represents that there are multiple multi-head self-attention modules.

**Figure 2:**
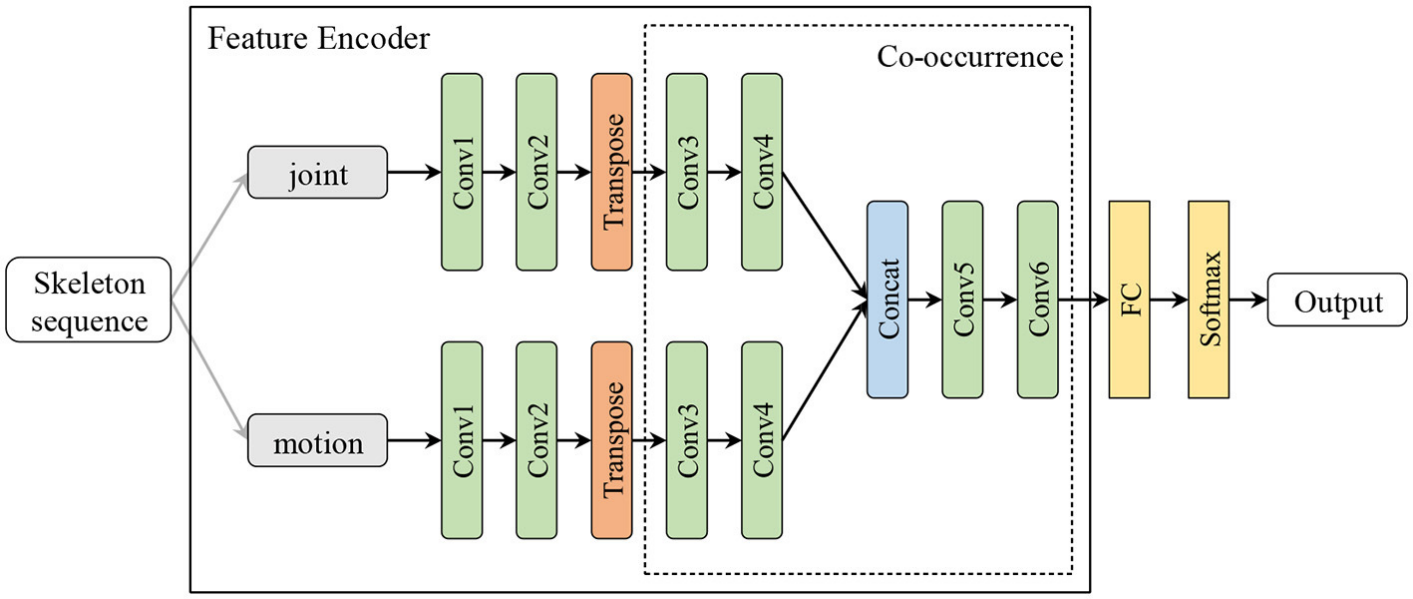
The feature encoder for skeleton sequence. The output channels of the 6 blocks 2D convolution layer are [64, 32, 32, 64, 128, 256]. The transpose layer transposes the dimensions of the input tensor according to the sequential parameters.

In order to obtain more effective features, we designed two feature encoders to handle IMU data and skeleton sequence, respectively, as shown in Figures 1, 2. In IMU data feature encoder, inspired by CSSHAR (Khaertdinov et al., 2021), we first employ a 1D convolution layer with 3 blocks for modeling in the temporal dimension, which includes a convolution kernel size of 3 and a feature map with channels of [32,64,128]. Furthermore, we employ a Transformer with a Multi-head self attention (heads *N* = 2) as the backbone to capture long-range dependencies from IMU data. Besides, inspired by hierarchical co-occurrence feature learning strategy, a two-stream framework is designed to learn and fuse the “joint” and “motion” features of skeleton sequences. Specifically, a skeleton sequence is divide into spatial joints and temporal motions. Then, they are fed into each of the four 2D CNN modules and assembled into semantic representations in both spatial and temporal domains, and point-level information of each joint is encoded independently.

### 3.3. Contrastive learning for unimodal recognition

**Figure 3:**
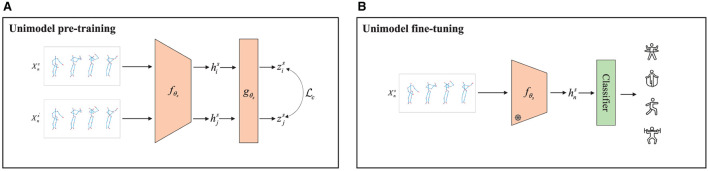
Contrastive learning framework for unimodal recognition. **(A)** Pre-training stage: for a skeleton sequence, the embedding representation *z* is generated by the same encoder *f* and projection head *g* after data augmentation using contrast loss *L*_*c*_, respectively. **(B)** Fine-tuning stage: the labeled skeleton sequence is passed through the frozen encoder *f*, and then processed through the classifier to obtain the action recognition label.

As shown in Figure 3, given a skeleton sample in the pre-training, a positive sample pair $X_{n}^{s}$ and $X_{n}^{s^{,}}$ could be obtained in a small batch by normal data augmentation. Then, they are fed into an encoder *f*_θ_*s*__ with HCN to yield the hidden layer features as


(1) $$\begin{array}{l} h_{i}^{s}=f_{\theta_{s}}\left(X_{n}^{s}\right) \end{array}$$

(2) $$\begin{array}{l} h_{j}^{s}=f_{\theta_{s}}\left(X_{n}^{s^{,}}\right) \end{array}$$

Inspired by the Barlow Twins, the feature representations $z_{i}^{s}$ and $z_{j}^{s}$ are obtained by an MLP projection layer, which are denoted as


(3) $$\begin{array}{l} z_{i}^{s}=g_{\theta_{s}}\left(h_{i}^{s}\right) \end{array}$$

(4) $$\begin{array}{l} z_{j}^{s}=g_{\theta_{s}}\left(h_{j}^{s}\right) \end{array}$$

Finally, to explore the relationship between the two views $X_{n}^{s}$ and $X_{n}^{s^{,}}$, the cross-correlation matrix C between embedding $z_{i}^{s}$ and $z_{j}^{s}$ can be computed as follows


(5) $$\begin{array}{l} C_{ij}=\frac{\sum_{b}z_{b,i}z_{b,j}^{\prime }}{\sqrt{\sum_{b}{\left(z_{b,i}\right)}^{2}}\sqrt{\sum_{b}{\left(z_{b,j}^{\prime }\right)}^{2}}}, \end{array}$$

where *b* denotes the batch dimension, *i* and *j* represent the embedding dimension. Finally, by enforcing the empirical cross-correlation matrix between the embeddings *Z*^*s*^ of variations to be an identity matrix, the encoder could be used to capture the relationship between the two-stream siamese networks. The contrastive loss function is formulated as follows


(6) $$L_{c}(Z^{s})=\sum_{i}{\left(1-C^{\prime }{}_{ii}\right)}^{2}+\beta \sum_{i}\sum_{j\neq i}C^{\prime 2}{}_{ij}$$

Intuitively, the first term encourages the diagonal elements of C to converge to 1, so that the embedding is not subject to variation. The second term is intended to drive the different embedding components to be independent of each other, minimizing the redundancy of the output units and avoiding becoming a constant. β is a positive constant used to weigh the first term and against the second term.

### 3.4. Contrastive learning for multimodal recognition

Our proposed VICReg-based multimodal recognition framework focuses on generating and contrasting embeddings from the IMU data and skeleton sequence branches, which eventually form a joint embedding architecture with variance, invariance and covariance regularization. It is a self-supervised learning method that incorporates two different modality training architectures based on the principle of preserving the content of the embedding information.

**Figure 4:**
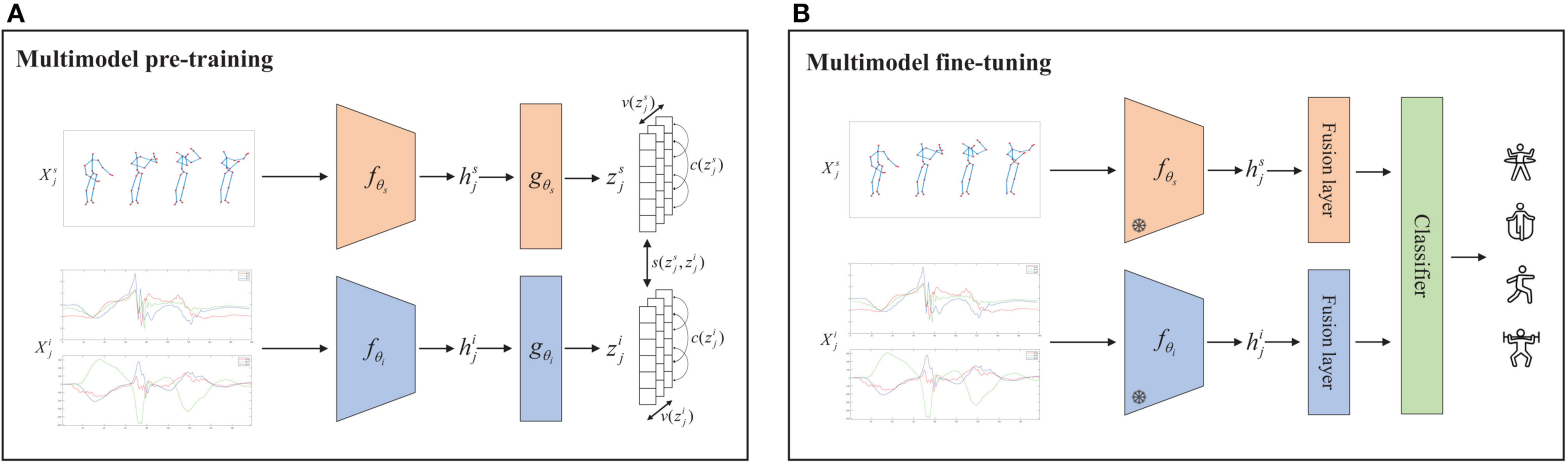
Contrastive learning framework for multimodal recognition. **(A)** Pre-training stage: the non-labeled skeleton sequences and IMU data were passed through modality-specific encoders *f* and projection heads *g* to generate embeddings representing *z* using contrasting loss. **(B)** Fine-tuning phase: the skeleton sequences with labels and the IMU data with labels were passed through frozen modality-specific encoders *f*, respectively, and then obtained action recognition label via fusion layer and classifier.

As shown in Figure 4, given a multimodal training sample {$x_{j}^{s},x_{j}^{i}$}, where *s* and *i* refer to skeleton and IMU data modalities respectively. The augmented inputs are generated by modality-specific data augmentation in accordance with


(7) $$\begin{array}{l} x_{j}^{s}=T\;\left(x_{j}^{s}\right) \end{array}$$

(8) $$\begin{array}{l} {\tilde{x}}_{j}^{i}=T\;\left(x_{j}^{i}\right) \end{array}$$

In details, for the skeleton sequence augmentation methods are jittering, scaling, rotation, shearing, cropping and resizing, whereas the IMU data augmentation methods are jittering, scaling, rotation, permutation, shuffle of channel. Then, the feature representation of the two modalities are computed. Specifically, two modality-specific encoders *f*_θ_*s*__ and *f*_θ_*i*__ perform feature extraction to obtain the high-dimensional hidden layer features.


(9) $$\begin{array}{l} h_{j}^{s}=\left(f_{\theta_{s}}\left({\tilde{x}}_{j}^{s}\right)\right) \end{array}$$

(10) $$\begin{array}{l} h_{j}^{i}=\left(f_{\theta_{s}}\left({\tilde{x}}_{j}^{i}\right)\right) \end{array}$$

Both of these are passed through projection heads *g*_θ_*s*__ and *g*_θ_*i*__, implemented by a multilayer perceptron, and finally generate mode-specific embeddings representations of the two modalities which are $z_{j}^{s}=g_{\theta_{s}}\left(h_{\theta_{i}}\right)$ and $z_{j}^{i}=g_{\theta_{i}}\left(h_{\theta_{i}}\right)$. The loss function is calculated at the embedding level with respect to $z_{j}^{s}$ and $z_{j}^{i}$. We describe the three components of variance, invariance and covariance that constitute our loss function in the pre-training process.

Firstly, we define the variance regularization term *v* to adopt the form of a hinge function that represents the standard deviation of the embeddings along the batch dimension.


(11) $$\begin{array}{l} v\left(Z\right)=\frac{1}{d}\overset{d}{\underset{j=1}{\sum }}\max\left(0,\gamma -Std\left(z^{j},\epsilon \right)\right), \end{array}$$

where *Std* denotes the regularization standard deviation formula as:


(12) $$\begin{array}{l} Std\left(x,\epsilon \right)=\sqrt{\mathrm{Var}\left(x\right)+\epsilon }, \end{array}$$

where we define *Z* = [*z*_1_, ..., *z*_*n*_] consisting of *n* vectors of dimension *d* with embeddings *z*_*j*_ from the feature encoding network of two modalities. *z*^*j*^ is represented as the value of each vectors in *Z* in dimension *j*, γ denotes a fixed value of the standard deviation and defaults to 1 in our experiments. ϵ is a small scalar to guarantee data stability, which is set to 0.0001. The objective of this regularization term *v*(*Z*) is to ensure that the variance of all embeddings *Z*^*s*^ and *Z*^*i*^ are close to γ in the current batch (*s* indicates the skeleton modality and i indicates the IMU modality), preventing all inputs from mapping on the same vector.

Secondly, we define the invariance regularization term *s* by using the mean square Euclidean distance between two positive sample pairs *Z*^*s*^ and *Z*^*i*^. The formulation is as follows:


(13) $$\begin{array}{l} s\left(Z^{s},Z^{i}\right)=\frac{1}{N}\overset{N}{\underset{j}{\sum }}∥z_{j}^{s}-z_{j}^{i}{∥}_{2}^{2}, \end{array}$$

where *N* denotes the batch size, both embeddings *Z*^*s*^ and *Z*^*i*^ come from the siamese architecture of the two branches.

Finally, the most critical component of the loss function, this term approximates the covariance between each pair of embedding variables to zero. Generally, it is the embeddings of the model that are decorrelated to each embedding variable to ensure the independence of the variables and prevent the model from learning similar or identical feature information. Inspired by Barlow Twins, we define the variance regularization term *c* as:


(14) $$\begin{array}{l} c\left(Z\right)=\frac{1}{d}\underset{i\neq j}{\sum }{\left[C\left(Z\right)\right]}_{i,j}^{2}, \end{array}$$

where the 1/*d* scales this function at the dimensional level and *C*(*Z*) denotes the covariance matrix of the embeddings *Z*. The formula is expressed as follows:


(15) $$\begin{array}{l} C\left(Z\right)=\frac{1}{N-1}\overset{n}{\underset{j=1}{\sum }}\left(z_{j}-\bar{z}\right){\left(z_{j}-\bar{z}\right)}^{T},\bar{z}=\frac{1}{N}\overset{N}{\underset{j}{\sum }}z_{j}. \end{array}$$

Therefore, the overall loss function with weighted average of the invariance, variance and covariance terms could be expressed as follows:


(16) $$\begin{array}{l} L\left(Z^{s},Z^{i}\right)=\lambda *s\left(Z^{s},Z^{i}\right)+\mu *\left[v\left(Z^{s}\right)+v\left(Z^{i}\right)\right]+\varphi *\left[c\left(Z^{s}\right)+c\left(Z^{i}\right)\right], \end{array}$$

where λ, μ, and φ are hyperparameters that measure the importance of each loss component. In our experiment, φ is set to 1 and a grid search is performed for the values of λ and φ with the basic condition λ = φ > 1.

The pseudo-code algorithm implementation is illustrated in Algorithm 1.

**Algorithm 1:**
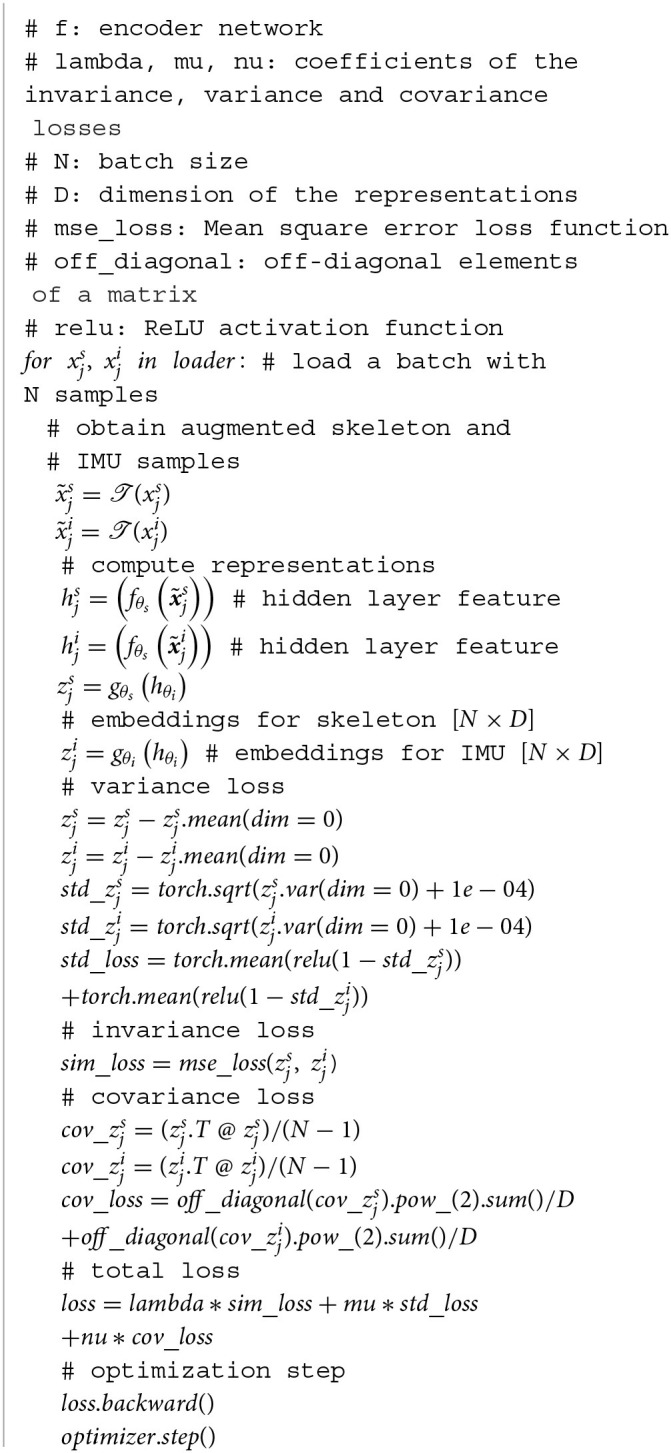
Multimodal pre-training pytorch pseudocode.

## 4. Experiments

### 4.1. Datasets

**UTD-MHAD** (Chen et al., 2015). The dataset is a multimodal dataset widely used for human action recognition, which includes RGB video, depth sequences, skeleton and IMU data. During the capturing process, 8 subjects perform 27 categories of actions, each individual repeating each action 4 times, for a total of 861 samples. For the skeleton sequences, the Kinect camera would capture information regarding the subject's posture and movements. For the IMU data, the subjects were required to wear gloves, shoes and belts with IMU sensors attached, which recorded motion information on the subject's body parts, including accelerations, angular velocities and gyroscope data. Similar to the evaluation protocol in the original paper, we use data from odd-numbered subjects: 1, 3, 5, 7 as the training and validation sets, and data from even-numbered subjects: 2, 4, 6, 8 as the testing set, and report the accuracy and F1 score on the testing set.

**MMACT** (Kong et al., 2019). The dataset is a multimodal dataset consisting of 20 subjects performing 36 classes of actions, including skeleton sequences and IMU data. In this work, a challenge version of the dataset with 2D keypoints is adopted for the skeleton data. The IMU data is derived from smartphones including accelerometers, gyroscopes and orientation sensors. We verify our proposed recognition framework against the evaluation protocol from the previous study: cross-subject and cross-scene. For the cross-subject setting, the first 16 subject samples are used for training and validation, while the remaining ones are used for testing. For the cross-scene setting, the numbered 2 samples from the occlusion scene were used for testing and the rest for training, numbered 1, 3, 4. We report the accuracy and F1 score on the testing set.

### 4.2. Implementations details

Our experimental environment is implemented on the A5000 GPU platform using the Pytorch framework. Subsequently, we detailed three aspects: data pre-processing, pre-training and fine-tuning.

**Data pre-processing**. In order to normalize the IMU data and skeleton sequences, we employed a resampling method to uniformly represent all sequences with 50 time steps. Furthermore, to ensure consistency and comparability, we applied a standard normalization procedure to normalize the joints in all skeleton sequences. This normalization process involved scaling the joint positions based on the reference frame established by the first frame of each sequence. For data augmentation of skeleton sequences, we employ {jittering, random resized crops, scaling, rotation, shearing} for two benchmarks. For data augmentation of IMU data, we employ {jittering, scaling, permutation, rotation, channel shuffle}.

**Pre-training**. For the UTD-MHAD dataset, in unimodal pretraining, our proposed method uses a batch size of 100 and sets the random seed for both skeleton and IMU modalities to 28. The training is performed for 100 epochs with a learning rate of 1e-2 and Adam optimizer. In the case of multimodal pretraining, our proposed method increases the batch size to 200 epochs, adjusts the learning rate to 1e-3, and sets the training scale to 200 epochs. The optimizer remains Adam. For the MMAct dataset, we maintain the same training settings as before, regardless of single or multi-modality. In unimodal pretraining, the learning rate is set to 1e-3, and the batch size is 96. In multimodal pretraining, we increase the batch size to 128 and adjust the learning rate to 1e-4. Similarly, the parameter initialization random seed is set to 28. All settings are shown in Table 1.

**Table 1:** Pre-training hyperparameter settings.

**Modality**	**UTD-MHAD**			**MMAct**		
	**Learning rate**	**Training scale**	**Batch size**	**Learning rate**	**Training scale**	**Batch size**
IMU	1e-2	100 epochs	128	1e-3	100 epochs	96
Skeleton	1e-2	100 epochs	128	1e-3	100 epochs	96
IMU+Skeleton	1e-3	200 epochs	256	1e-4	200 epochs	128

**Fine-tuning**. Following prior fine-tuning routines, we implemented modality-specific feature fusion layers for the multimodal fine-tuning process, including batch normalization and non-linear ReLU, mapping the embeddings of IMU data and skeleton sequence to the same size of 256. And then concatenated them up by a linear classifier with Softmax function. We train the samples with labels by fine-tuning the model both to 100 epochs either unimodal or multimodal for our action recognition task.

### 4.3. Evaluations

#### 4.3.1. Learning feature representation

To evaluate the multimodal learned feature representation, we perform linear evaluation of the features extracted from a specific encoder and then input the labeled samples into the fine-tuned training encoder and linear classifier. The performance of our model is compared with existing state of the art methods, and the results as shown in Table 2.

**Table 2:** The performance of action recognition for accuracy (%) and F1 score (%) is compared with the baseline methods.

**Method**	**Modality**	**UTD-MHAD**		**MMAct cross-subject**		**MMAct cross-scene**	
		**Accuracy**	**F1-score**	**Accuracy**	**F1-score**	**Accuracy**	**F1-score**
Supervised_transformer	IMU	79.77	79.59	62.15	62.32	78.27	71.86
Supervised_cooccurrence	Skeleton	93.49	93.43	80.53	81.93	78.61	74.30
SimCLR	IMU	64.65	64.64	52.32	51.94	66.16	60.28
SimCLR	Skeleton	92.09	91.87	75.97	76.75	72.62	62.04
Barlow Twins	IMU	58.60	57.69	45.17	44.11	59.96	51.77
Barlow Twins	Skeleton	88.84	88.24	67.86	69.24	60.68	52.34
Barlow Twins	IMU+Skeleton	91.63	91.72	82.17	81.98	82.70	80.05
CMC	IMU+Skeleton	95.12	95.08	82.05	83.06	84.01	82.41
CMC-CMKM^§^	IMU+Skeleton	95.81	95.74	82.34	82.69	85.24	83.60
**Ours**	IMU	75.58	75.93	49.04	47.08	60.81	53.80
**Ours**	Skeleton	86.05	86.23	73.78	75.66	74.94	73.29
**Ours**	IMU+Skeleton	96.06	**96.96**	**82.95**	**83.62**	87.06	85.78
Supervised	IMU+Skeleton	**96.51**	96.36	81.78	82.86	**89.47**	**87.94**

Bolded data indicate the best results, underlined data the second best. § represents the reproduced results.

From the accuracy and F1 score terms obtained from the linear evaluation, our method significantly outperforms unimodal (more than 20% for IMU and almost 10% for Skeleon) for two benchmarks when multimodal contrastive learning is implemented. When comparing the self-supervised learning baseline models, our method is superior to other contrastive learning methods in terms of the multimodal learning approach. However, for the unimodal learning approach, our method has relatively no advantage. It is possible that our method undergoes a certain degree of embeddings collapse when calculating the standard deviation and variance. Meanwhile, the accuracy and F1 score of our method are also slightly lower when comparing fully supervised learning, which may be due to the fact that the supervised learning approach can perform end-to-end feature extraction for specific modalities. It is worth noting that our proposed method achieves 82.95% accuracy and 83.62% F1 score for MMAct (cross-subject), which exceeds the supervised learning method by 1.17 and 0.76%, indicating that our method has a better learned feature representation for multimodal training.

#### 4.3.2. Semi-supervised learning

In the experiments, we adopt proportional unlabeled IMU and Skeleton data to perform contrastive learning in the pre-training phase. In particular, we set a random percentage *p*∈{1%, 5%, 10%, 25%, 50%} to conduct the experiment. To obtain a reasonable fine-tuning result, we calculate the average accuracy under the evaluation protocol corresponding to that presented in the colored interval by repeating the training 10 times on each *p*. In addition, we train a supervised learning multimodal model using the same encoders (Transformer for IMU and Co-occurrence for Skeleton). Similarly, fine-tuning the two-stream siamese networks and performing feature fusion, the final recognition results are obtained by a linear classifier, especially noting that the weights of the encoders are randomly initialized.

**Figure 5:**
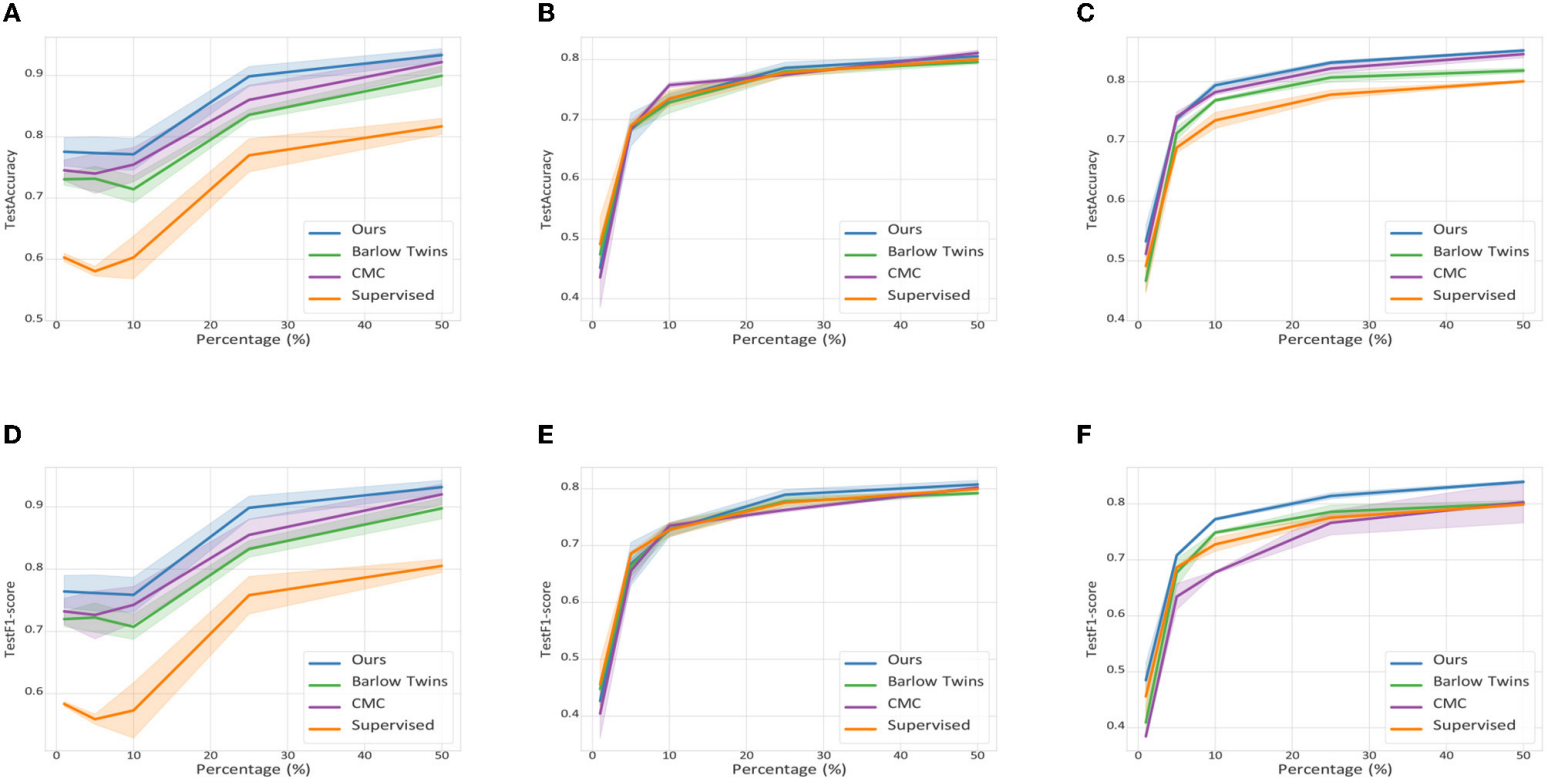
Average accuracy and F1 score with 95% confidence intervals for the semi-supervised learning scenario. **(A)** UTD-MHAD. **(B)** MMAct (cross-subject). **(C)** MMAct (cross-scene). **(D)** UTD-MHAD. **(E)** MMAct (cross-subject). **(F)** MMAct (cross-scene).

As shown in Figure 5, despite training only a small number of labeled samples, the contrastive learning methods all exhibit excellent robustness and performance. Specifically, the contrastive learning based approach outperforms the supervised learning based approach when the labeled samples are less than 25%, regardless of the dataset. Besides, our proposed method is superior to both Barlow Twins and CMC contrastive learning based multimodal methods with arbitrary *p* values, which further validate the effectiveness and generalization ability of our proposed method.

#### 4.3.3. Qualitative analysis

In order to evaluate the clustering effect of the model from a qualitative perspective, we employ a t-Distributed Stochastic Neighbor Embedding (t-SNE, van der Maaten and Hinton, 2008) method to visualize the high-dimensional embeddings into a two-dimensional plane.

**Figure 6:**
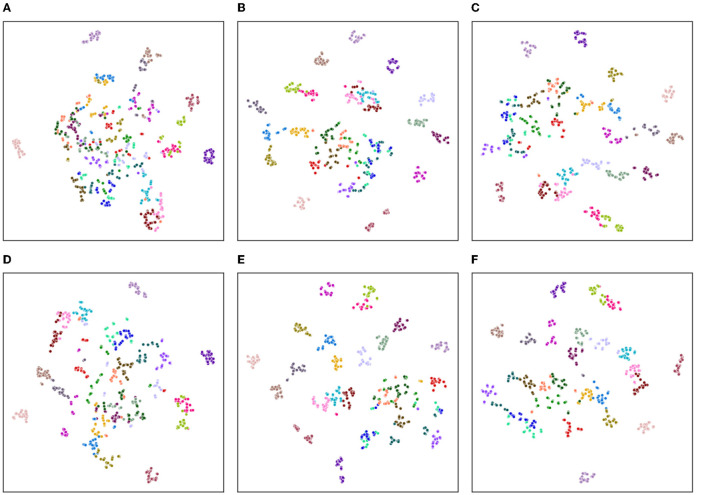
Visualization of representations learned using t-SNE for the UTD-MHAD benchmark. **(A)** Barlow Twins (IMU). **(B)** Barlow Twins (Skeleton). **(C)** Barlow Twins (IMU+Skeleton). **(D)** Ours (IMU). **(E)** Ours (Skeleton). **(F)** Ours (IMU+Skeleton).

**Figure 7:**
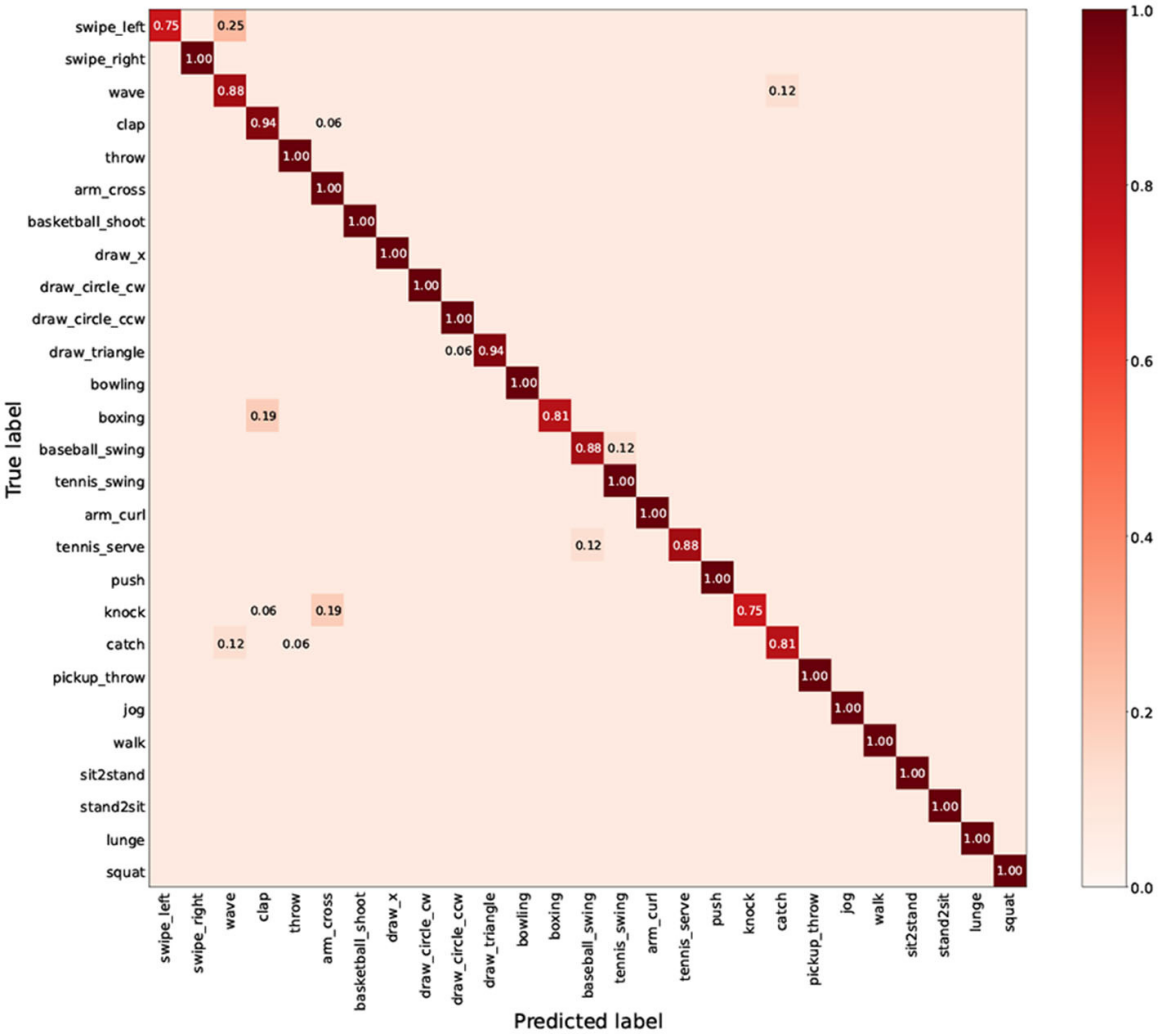
The normalized confusion matrix for UTD-MHAD.

**Figure 8:**
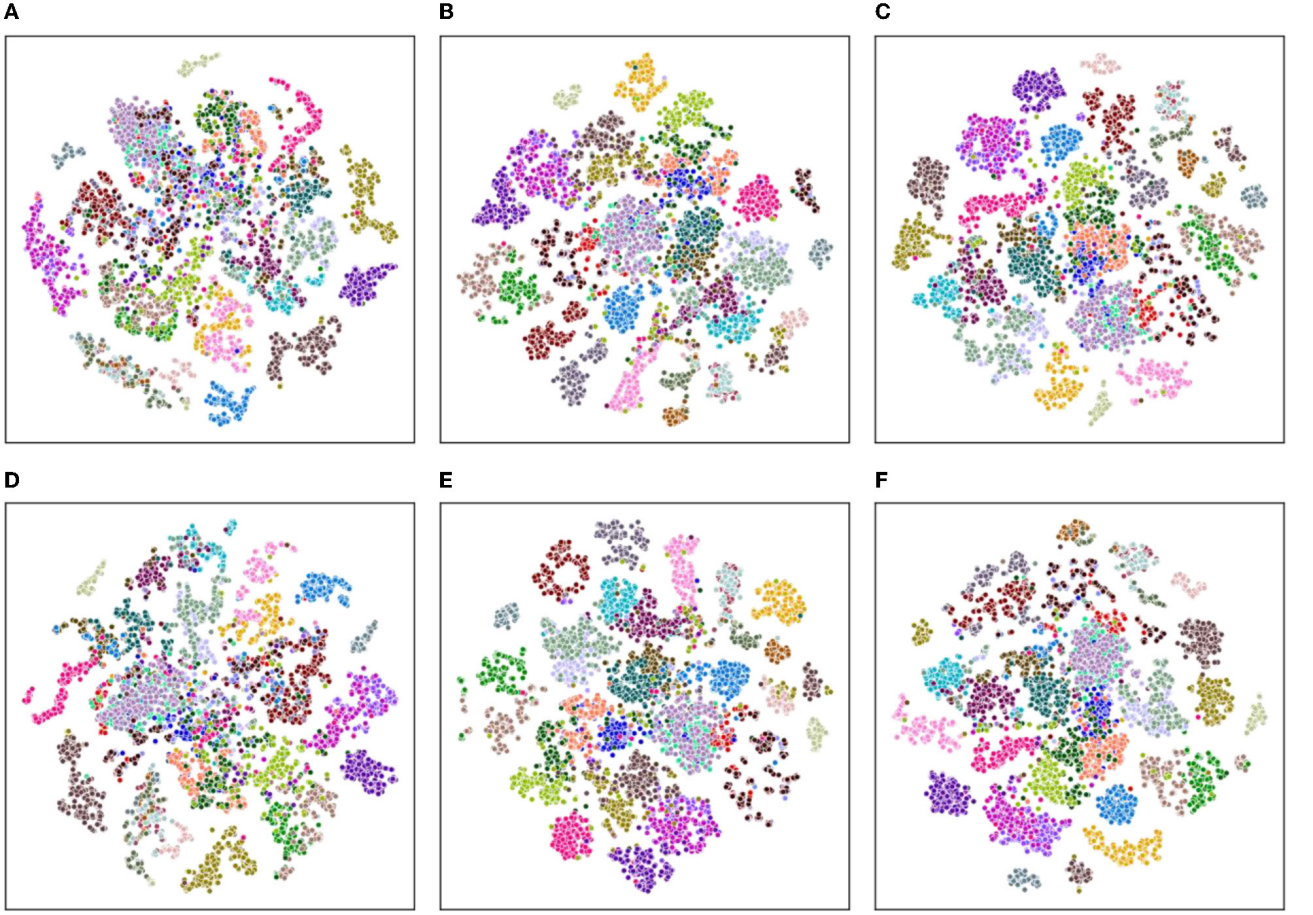
Visualization of representations learned using t-SNE for the MMAct benchmark. **(A)** Barlow Twins (IMU). **(B)** Barlow Twins (Skeleton). **(C)** Barlow Twins (IMU+Skeleton). **(D)** Ours (IMU). **(E)** Ours (Skeleton). **(F)** Ours (IMU+Skeleton).

**Figure 9:**
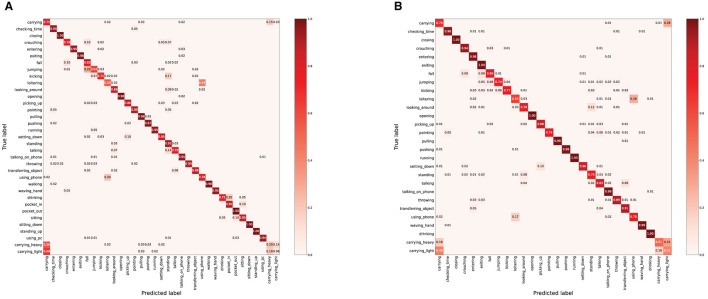
The normalized confusion matrix for MMAct benchmarks. **(A)** MMAct (cross-subject). **(B)** MMAct (cross-scene).

As shown in Figures 6, 7, we explore the IMU-based, Skeleton-based and multimodal approaches on the UTD-MHAD and MMAct datasets, respectively. Compared to the Barlow Twins, from an intuitive point of view, our proposed method is obviously effective in separating action class. Moreover, it is discovered that the multimodal data clustering is better than the unimodal clustering by fusing the features of IMU and Skeleton modalities. Furthermore, to measure the classification performance of our proposed method after fine-tuning, we performed accuracy evaluation by normalizing the confusion matrix. As shown in Figures 8, 9, we plot the normalized confusion matrices on UTD-MHAD, MMAct (cross-subject) and MMAct (cross-scene) to intuitively evaluate the performance of the classifier.

### 4.4. Zero shot setting

In the zero shot setting, we further explore the proposed method on the IMU and skeleton modalities through hiding certain action groups during the pre-training process. Specifically, we ensured that the action categories index [1, 2, 5] were not leaked during the training process by masking them.

As shown in Tables 3, 4, the performance of our model is compared with existing state of the art methods. Regarding UTD-MHAD benchmark for the unimodal evaluation, we could observe that the difference of the model is not significant after fine-tuning, but the skeleton sequence-based is much higher 15% than the IMU-based method. This is probably due to the fact that the skeleton sequences are modeled in both spatial and temporal dimensions, whereas IMU is only considered in the temporal dimension. For the multimodal evaluation, the model achieved 96.05% for accuracy and 96.00% for F1 score with class_id = < 5> hidden, which is very close to the results achieved without the zero shot approach. Furthermore, regardless of the action class hidden, it is noted that the multimodal-based achieves much higher accuracy than the unimodal-based approach, exceeding the IMU-based approach by approximately 20% and the skeleton-based approach by approximately 6%. This validates that our proposed method achieves superior results with multimodal data inputs, which demonstrate the ability of the proposed method to learn complementary information.

**Table 3:** Zero shot performance (%) on UTD-MHAD benchmark.

**Modality**	**num_classes=1**		**num_classes=2**		**num_classes=5**	
	**Accuracy**	**F1-score**	**Accuracy**	**F1-score**	**Accuracy**	**F1-score**
IMU	73.95	73.95	73.49	73.79	75.35	75.54
Skeleton	88.84	88.43	87.91	87.84	89.77	89.51
IMU+Skeleton	95.58	95.59	93.95	93.85	96.05	96.00

**Table 4:** Zero shot performance (%) on MMAct benchmark.

**Modality**	**num_classes=1**		**num_classes=2**		**num_classes=5**	
	**Accuracy**	**F1-score**	**Accuracy**	**F1-score**	**Accuracy**	**F1-score**
IMU	48.39	48.11	48.31	47.34	48.81	48.63
Skeleton	73.67	75.74	72.35	73.97	73.68	75.83
IMU+Skeleton	81.39	81.19	81.73	82.35	82.45	83.02

## 5. Conclusion

In this paper, we propose a simple and effective contrastive self-supervised learning framework for human action recognition. Specifically, we construct a multimodal dataset by combining skeleton sequences and IMU signal data, and feed them into pretrained modality-specific two-stream networks for feature encoding. During the fine-tuning stage, labeled data is fed into the frozen encoders with weight initialization, and a linear classifier is applied to predict actions. Extensive experiments demonstrate that our proposed method outperforms unimodal approaches. It is worth noting that our model achieves comparable performance to pure supervised multimodal learning in certain metrics. In the future, we plan to further investigate other modalities, such as depth maps and RGB videos, to enhance multimodal human action recognition methods. Additionally, by incorporating knowledge distillation and unsupervised learning techniques, we aim to explore different ways of feature fusion between modalities to improve its performance in complex scenarios.

## Data availability statement

The original contributions presented in the study are included in the article/supplementary material, further inquiries can be directed to the corresponding author.

## Author contributions

ZR and HYu: conceptualization and writing—review and editing. HYa: methodology and validation. ZR and ZX: software. JZ: formal analysis. HYu: resources. ZX and JZ: data curation and visualization. HYa and ZR: writing—original draft preparation. ZR: supervision and funding acquisition. All authors contributed to the article and approved the submitted version.
